# Antibacterial and Antioxidant Activities of Triterpenoids Isolated from Endemic *Euphorbia arbuscula* Stem Latex

**DOI:** 10.1155/2024/8273789

**Published:** 2024-03-09

**Authors:** Zainab Al-Ansi, Mohammed Masaoud, Khaled Hussein, Bushra Moharram, Wafa M. Al-Madhagi

**Affiliations:** ^1^Department of Organic Chemistry, Faculty of Science, Sana'a University, Sana'a, Yemen; ^2^Research Center, Faculty of Pharmacy, Sana'a University, Sana'a, Yemen; ^3^Department Pharmacognosy, Faculty of Pharmacy, Sana'a University, Sana'a, Yemen; ^4^Department of Pharmacy, Faculty of Medical Science, Al Nasser University, Sana'a, Yemen; ^5^Department of Pharmaceutical Chemistry, Faculty of Pharmacy, Sana'a University, Sana'a, Yemen

## Abstract

This research study aimed to investigate the chemical constituents and evaluate the antibacterial and antioxidant activities of stem latex extracts from the endemic medicinal plant *Euphorbia arbuscula* found on Socotra Island, Yemen. The study aimed to assess the potential medicinal and veterinary uses of this plant, representing the first evaluation of its properties. The stem latex was extracted using ethanol, and the resulting oil underwent analysis using GC-MS to identify eight compounds. In addition, chromatographic techniques were employed to isolate two triterpenoids, lanosterol and lupeol, from the stem latex. The structures of these compounds were confirmed using IR, MS, and NMR techniques. The antibacterial activity of the extracts and isolated compounds was evaluated against three bacterial strains using the disc diffusion method, revealing only weak antibacterial effects. The study also investigated the antioxidant activity using the DPPH assay, where the ethyl acetate extract exhibited the highest activity with an IC_50_ value of ±13.55 *µ*g/mL, followed by the chloroform extract with an IC_50_ of ±21.87 *µ*g/mL. These findings emphasize the potential of *Euphorbia arbuscula* in the development of new medicines, particularly due to its notable antioxidant activity. The research methodology employed a scientifically rigorous approach, utilizing a comprehensive range of analytical techniques. However, further investigation is required to fully assess the plant's potential as a therapeutic agent.

## 1. Introduction

The Euphorbiaceae plant family holds significant importance in Yemen, consisting of 106 species, with 62 species belonging to the genus Euphorbia, out of which 16 are endemic [1, 2]. Notably, 11 of these endemic species are exclusively found on the island of Socotra. Extensive research has been conducted on various parts of *Euphorbia* plants, including roots, seeds, latex, lactiferous tubes, stem wood, stem barks, leaves, and whole plants, to explore their chemical compositions and biological activities.

Triterpene alcohols present in *Euphorbia* latex have been identified as useful chemotaxonomic markers [3, 4]. For instance, compounds such as 24-methyl-9,19-cyclolanost-24-en-3-ol and 19-norlanosta-5,24-dien-3-ol,9-methyl-,(3*β*,9*β*,10*α*) were discovered in *E. caducifolia* latex extract [5]. In addition, cyclotirucanenol and cycloeuphordenol were isolated from *E. tirucalli* [6, 7] latex, while 9,19-cyclolanost-22(22′),24-diene-3-ol (nerifoliene) and euphol were obtained from fresh latex of latex *E. nerifolia* [8]. These compounds play a crucial role in the identification and classification of *Euphorbia* species.

*Euphorbia* species have a long history of traditional use in various countries worldwide for the treatment of diverse ailments such as cancer, diabetes, heart diseases, cough, earache, and rheumatism [4, 9]. The latex of *Euphorbia* plants contains triterpenoid alcohols, including tigliane diterpenoids, ingenol, phorbol esters, and tirucallol. Tirucallol has demonstrated anti-inflammatory and antiparasitic properties, while cycloartenol, another triterpenoid alcohol found in Euphorbia latex, exhibits potential anticancer activity.

*Euphorbia arbuscula*, commonly known as Amtech, is an endemic plant species found exclusively on the island of Socotra. The traditional uses of its latex include cauterizing or sealing wounds, alleviating breathing difficulties, repelling flies, and providing bandages for sprains or fractures. Furthermore, the latex of *Euphorbia arbuscula* is utilized as an adhesive and is considered the strongest available on Socotra.

Given the significance of the traditional uses of Euphorbia species, particularly *Euphorbia arbuscula*, in wound healing and fly repellency, this study aims to isolate and identify chemical compounds present in its stem latex. By investigating the chemical constituents of *Euphorbia arbuscula*, this research seeks to enhance our understanding of its medicinal potential and explore new therapeutic applications.

## 2. Material and Methods

### 2.1. Plant Material

The fresh stem latex of *E. arbuscula* was collected from Socotra Island, Yemen, in October and December 2014, and the fresh latex was weighed 894 g. The plant material was identified by Dr. Abdul Wali Al-Khulaidi (Faculty of Science, Taiz University, the Agricultural Research and Extension Authority, Yemen) and Abdul Habib Al-Qadasi (the Agricultural Research and Extension Authority, Yemen). Its voucher specimen (No. YP2496) was deposited in the Socotra Herbarium.

### 2.2. Plant Preparation and Extraction of Stem Latex

**Figure 1:**
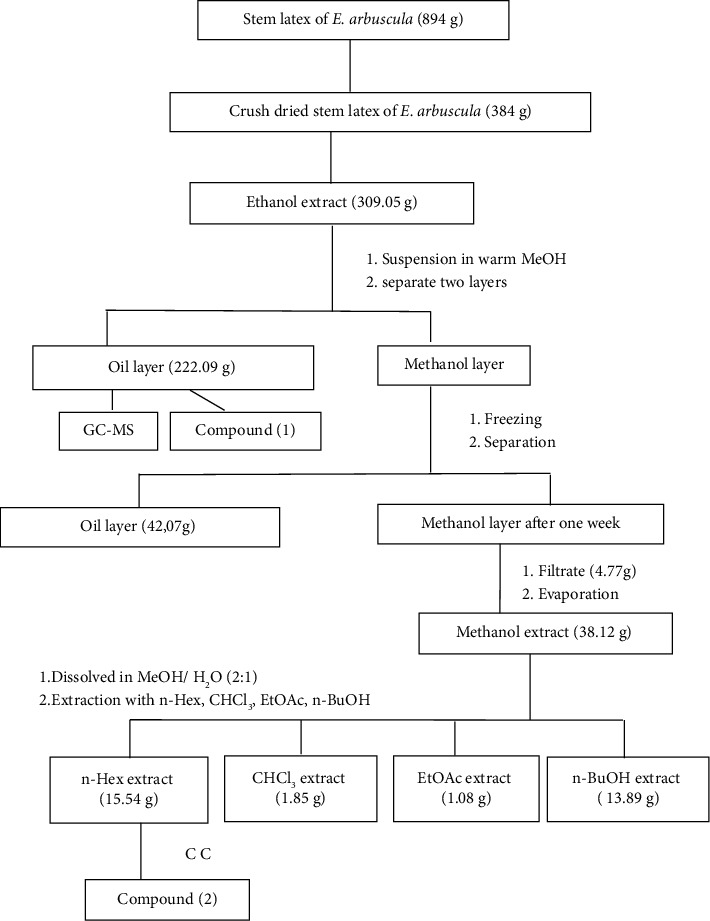
Flowchart of extracts and fractionations of stem latex *E. arbuscula*: methanol (MeOH), n-hexane (n-Hex), chloroform (CHCl_3_), ethyl acetate (EtOAc), and n-butanol (n-BuOH).

The stem latex of *E. arbuscula* was dried under shade for one week. The crushed dried stem latex (384 g) was macerated in 90% ethanol (90 ethanol: 10 water; (6 × 1 L)) with shaking for two days at room temperature. The combined extracts were filtered and evaporated. The ethanolic dried extract (309.05 g) was suspended in 600 mL of warm methanol to give two layers; the lower layer was oily (257 mL, (222.09 g)) while the upper layer was methanol. The methanol layer was frozen for two days (to remove the rest of the oil) to give two layers; the lower layer was separated (48.31 mL, (42.07 g)). The methanol layer after one weak at room temperature gave a precipitate (4.77 g) which is separated by filtration and then evaporated to give 38.12 g. The dried methanol extract was dissolved in methanol: water (2 : 1) and then fractionated successively with n-hexane (3 × 500 mL), chloroform (3 × 500 mL), ethyl acetate (6 × 500 mL), and finally butanol (3 × 500 mL). Each fraction was dried, yielding four fractions of 15.54, 1.85, 1.08 g, and 13.89 g, respectively. At the same time, the first oily layer was isolated and identified by GC-MS. The overall process of stem latex extraction of *E. arbuscula* is summarized in the flowchart in Figure 1.

### 2.3. Phytochemical Analysis

Phytochemical screening of the stem latex extracts was performed using standard methods to identify various phytochemicals, including terpenoids, alkaloids, flavonoids, tannins, phenols, glycosides, cardiac glycosides, steroids, and saponins [10, 11].

### 2.4. Isolation of Constituents

Compound **(1)** was isolated for the first time by sample extraction from the first oily layer by dissolving 50 g of the oily extract in 30 mL methanol. Dissolved oil was reduced to half by rotary evaporator, n-hexane was added to dissolved oil, and then n-hexane layer gave white powder (5.57 g).

Compound **(2)** was isolated from n-hexane extract by suspension in n-hexane and then filtered and evaporated. The resulting dried n-hexane extract (10.62 g) was fractionated using column chromatography with n-hexane: chloroform (10:0 to 0:10), chloroform: ethyl acetate (10:0 to 0:10), and ethyl acetate: methanol (10:0 to 0:10) as eluents. TLC indicated differences in composition, and five fractions (HE1-HE5) were obtained. Fraction HE2 (6.0 g) was further fractionated using column chromatography with n-hexane: chloroform (10:0 to 0:10), chloroform: ethyl acetate (10:0 to 0:10), and ethyl acetate: methanol (10:0 to 0:10) as eluents. The resulting five fractions (HE2F1-HE2F5) were collected. Fraction HE2F3 (0.53 g) was crystallized from methanol to afford white crystals, which yielded (100 mg).

### 2.5. Identification of Compounds

All chemicals and solvents were obtained from Sigma-Aldrich (Germany) and Scharlau (Spain). The Buchi Rotavapor R-200 system with Buchi water bath B-490 and Buchi V-800 vacuum (Germany) was used. TLC spots were visualized under UV light at 254 nm and 365 nm using VIBER-Lourmat equipment (French), and IR spectra were obtained using the potassium bromide disc technique on a JASCO 410 FT-IR.

### 2.6. Gas Chromatography-Mass Spectroscopy

Gas chromatography-mass spectrometry (GC-MS) was performed using a Gas Chromatography-Mass Spectrometer GCMS QP2010 Plus (Shimadzu, Japan) equipped with an autosampler. The sample, dissolved in GC-MS grade chloroform, was injected (1 *μ*L) into a fused silica capillary column HP5-MS (30 m × 0.32 mm, film thickness 0.25 *μ*m) with a split ratio of 1/50 and helium as the carrier gas. The oven temperature was initially held at 50°C for 5 min, then increased at a rate of 5°C per minute to 240°C, and maintained at 240°C for 5 min. The final temperature was 250°C. The components of the test oil were identified by comparing their spectra with those of known compounds stored in the internal library (Wiley; mass spectral library) using a mass spectrometer that was operated in the electron ionization (EI) mode with a beam energy voltage of 70 eV with molecular weight range 30–600 m/z.

Nuclear magnetic resonance (NMR) spectra were recorded using a Bruker spectrometer in CDCl3 as the solvent. The spectra were obtained at 400 MHz (1H) and 100 MHz (^13^C).

Pure compounds were isolated using various chromatographic techniques, including thin-layer chromatography (TLC) on aluminum sheets (20 × 20 cm) coated with 60 GF254 (Merck, Germany) and column chromatography (CC) (50 × 2.5 cm) on a silica gel mesh (0.063–0.200 mm) (Merck, Germany).

### 2.7. Biological Activities

The extracts of stem latex of *E. arbuscula*, n-hexane, chloroform, ethyl acetate, butanol, methanol, and ethanol, as well as the isolated pure compounds (1,2), were evaluated for antibacterial and antioxidant activities.

### 2.8. Antibacterial Activity

The antibacterial activity was evaluated by disc diffusion method [12, 13]. Bacterial stock cultures were obtained from the Yemeni Pharmacovigilance Center, including *Staphylococcus aureus* (ATCC 6538), *Escherichia coli* (ATCC 8739), and *Pseudomonas aeruginosa* (ATCC 25619). A stock solution of all samples (500 mg/ml) was prepared in methanol (99.8%). Sterile paper discs (Whatman NO3) with a diameter of 6 mm were impregnated with 15 *μ*l of the samples (500 mg/ml) and air-dried under sterile conditions. Mueller Hinton agar (MHA) (90 mm) was inoculated with the stock bacterial suspension (1-2 × 108 CFU/ml) by streaking the surface in three different directions to ensure proper distribution of the inoculum with a sterile cotton swab and incubating of the plates at 37°C for 24 h. The antibacterial activities were determined by measuring the diameter of the inhibition zone for each extract and compound against positive controls (30 *μ*g/disc) of gentamicin; 15 *μ*l of the methanol was used as a negative control. The mean inhibition zone diameter was recorded from triplicate tests, and the antibacterial evaluation was defined as strong if the inhibition zone diameter was >15 mm, moderate for diameters between 10 and 15 mm, and weak for diameters >10 mm [14].

### 2.9. Antioxidant Activity

The antibacterial activity was evaluated by measuring their free radical scavenging activity using DPPH (1,1-diphenyl-2-picrylhydrazyl) [13, 15]. Various concentrations of the stock solution (10 mg/ml) of extracts and isolated compounds of stem latex *E. arbuscual* were prepared in methanol (1000, 500, 250, 125, 62.5, 31.25, 15.6, and 7.8 *μ*g/mL). A serial dilution of stock solution (10 mg\mL) of ascorbic acid was also prepared in methanol to get concentrations ranging between 100 and 3.12 *μ*g/mL as a positive control. DPPH was prepared freshly by dissolving 0.6 mg of 1,1-diphenyl-2-picrylhydrazy in about 15 mL of methanol to produce 0.1 mM. Quantitative estimation was carried out in 96 wells plate, and 100 *μ*l of tested samples dissolved were added to each well and mixed with 100 *μ*l of DPPH in methanol (0.1 mM). The negative control sample containing 100 *μ*L of DPPH was used in the last row of the plate. The test samples without DPPH were also used as the blank. The mixture was shaken for 1 min to be homogenous and stored in the dark. After 30 minutes of incubation at 25°C, the mixture was measured at wavelength 492 nm by using a multiscan spectrum instrument, and the experiment was done in triplicate. Radical scavenging activity was calculated using the following formula:

(1) $$\begin{array}{c} \%\;\text{of}\;\text{inhibition}=\left[\left(\text{AC}–\frac{\left(\text{AE}-\text{AB}\right)}{\text{AC}}\right]\right.\times 100\%, \end{array}$$

where AC is the absorbance of the negative control, AE is the absorbance of the test sample with DPPH, and AB is the absorbance of the blank. The correlation between each concentration and its percentage of free radical scavenging was plotted, and the IC_50_ was calculated by interpolation. The activity was expressed as IC_50_, the inhibition concentration of each extract that scavenges 50% of DPPH radicals.

## 3. Results

**Figure 2:**
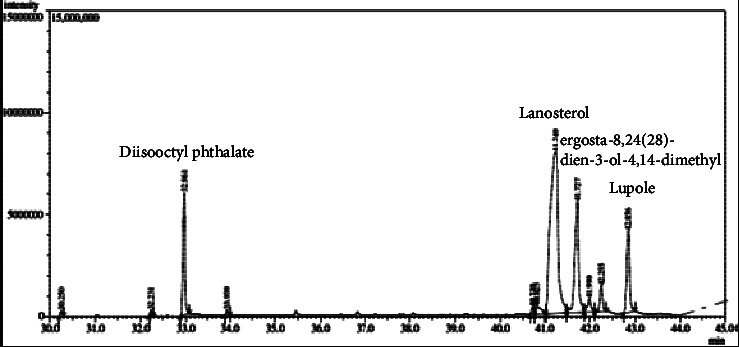
GC-MS chromatogram of oil extract of the stem latex *E. arbuscula*.

The yield of stem latex of *Euphorbia arbuscula* extracts is presented in Table 1. The percentage of crude ethanol extract obtained from dry weight of the stem latex of *E. arbuscula* was 80.48%. The highest percentage obtained from the crude ethanol extract was the first extraction of the oil (57.83; 71.86%), while the lowest was obtained from ethylacetate extract (0.28; 0.34%). The results obtained from the phytochemical screening of stem latex of *E. arbuscula* extracts are presented in Table 2. Phytochemical screening ensured the presence of tanin and phenols in the tested extracts. Steroids and terpenoids were found in the methanol, n-hexane, and chloroform extracts. The alkaloids, cardiac glycosides, and flavonoids were selectively distributed in all latex extracts, while glycosides were absent in all extracts. The first oily layer was isolated from methanol identified by GC-MS. The phytocomponents were identified by comparing their mass spectra with those in mass spectral libraries (Wiley; mass spectral library) to ascertain their name, molecular weight, and structure.  Figure 2 shows the total ion chromatogram provided details of peak number, the retention time (RT), name of the compound, molecular formula, molecular weight (MW), and area percentage presented in Table 3. At the same time, compound **(1)** was isolated from the first oil layer as a white powder dissolved in CHCl_3_; Rf, 0.73 with EtOAc: n-Hex (7 : 3) and indicated by spray reagent 5% sulfuric acid as a brown zone in visible. The melting point was 135–137°C. The spectral data of the compound were as follows: IR (KBr): *ν*max cm^−1^ 3440, 2950, 2880, 1680, 1460, 1390, and 1050 cm^−1^. The MS spectra are obtained at 70 eV for C_30_ H_50_O m/z relative intensity of molecular ion (rel. int.%): [M^+^] 426 (35%), 411 (100%), 393 (62%), 259 109 (60%), ^1^H NMR (CDCl_3_, 400 MHz) *δ* (ppm): 0.75 (s, ^3^H, 18-H), 0.80 (s, ^3^H, 29-H), 0.85 (s, ^3^H, 30-H), 0.88 (d, *J* = 4, ^3^H, 21-H), 0.95 (s, ^3^H, 19-H), 1.00 (s, ^3^H, 28-H), 1.60 (s, ^3^H, 27-H), 1.68 (s, ^3^H, 26-H), 3.25, 3.22 (dd, *J* = 4 and 12, ^1^H, H_−3_), 5.11 (t, *J* = 8, ^1^H, 24-H); 13C NMR (CDCl_3_, 100 MHz) *δ* (ppm) shown in Table 4, while compound **(2)** was obtained by fractionating n-hexane using column chromatography as a white crystal dissolved in CHCl_3_; Rf, 0.81 with CHCl_3_: MeOH (9.5 : 0.5) and indicated as violet zone by vanillin sulfuric acid reagent. The melting point was 215–217°C. The spectral data of the compound were as follows: IR (KBr): *ν*max cm^−1^ 3397, 2941, 2857, 1638, 1454, 1379, and 1043 cm^−1^. The MS spectra are obtained at 70 eV, for C_30_H_50_O m/z relative intensity of molecular ion (rel. int.%): 426 [M+] (35%), 411 (19.7%), 207 (50.3%), 189 (70%), 175 (30.2%), 121 (75%), and 95 (99%). ^1^H NMR (CDCl_3_,400 MHz) *δ* (ppm): 0.72 (s, ^3^H, 24-H), 0.76 (s, ^3^H, 28-H), 0.88 (s, ^3^H, 25-H), 0.90 (s, ^3^H, 27-H), 0.96 (s, ^3^H, 23-H), 1.32 (s, ^3^H, 26-H), 1.61 (s, ^3^H, _30-H_), 2.36 (m, ^1^H, H_−19_), 3.10, 3.12 (dd, *J* = 4.1 and 10.9, ^1^H, H_−3_), 4.50 (d, *J* = 1.4, ^1^H, H_-29a_) and 4.62 (d, *J* = 1.4, ^1^H, H-29b). ^13^C NMR (CDCl_3_, 100 MHz) *δ* (ppm) is shown Table 4.

### 3.1. Determination of the Antibacterial Activity

The antibacterial activity extracts and pure compounds isolated from the stem latex of *E. arbuscula* were evaluated against *S. aureus*, *E. coli*, and *P. aeruginosa* using the disc diffusion method. The tested extracts and isolated compounds showed weak and no antibacterial activity.

### 3.2. Determination of Antioxidant Activity

**Figure 3:**
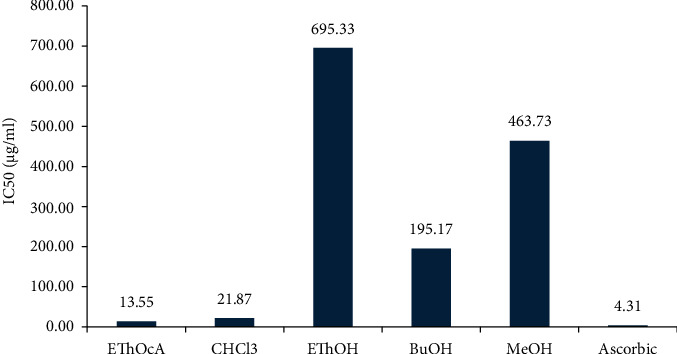
IC_50_ values (*µ*g/ml) of various latex extracts of *E. arbuscula* and compared to vitamin c.

The extracts and isolated compounds showed dose-dependent activity, where the % inhibition increased as the concentration of the extract or compounds increased. The ethyl acetate extract exhibited the highest activity with an IC_50_ value of 13.55 *μ*g/mL, which was comparable to ascorbic acid (IC_50_ 4.09 *μ*g/mL) followed by chloroform extract with an IC_50_ value of 21.87 *μ*g/mL, as illustrated in Figure 3.

## 4. Discussion

The GC-MS analysis of the first oil layer obtained from the latex extract of *E. arbuscula* revealed the presence of eight compounds, as shown in Table 3. The major compounds identified were lanosterol (51.98%), ergosta-8,24(28)-dien-3-ol-4,14-dimethyl (17.82%), dioctyl phthalate (9.93%), and lupeol (9.86%). Of particular interest was the identification of lanosta-8,24-diene-3-one, which has not been previously reported in the Euphorbiacea family. Furthermore, the GC-MS analysis indicated the presence of a rich diversity of triterpenoids in the oil layer. These findings are consistent with previous studies on other Euphorbia species, which have been reported to contain various triterpenoids in their latex and other plant parts.

Compound **(1)**: The melting point (135–137°C) was in agreement with the previously published data of Lanosterol [16]. The compound showed a brown spot visible on TLC when it was sprayed with 5% H_2_SO_4_ indicating that the compound could be a sterol [17]. The IR spectrum showed a broad strong band at 3440 cm^−1^ indicating the presence of OH while two bands at 2950 and 2880 cm^−1^ (C-H stretching) for CH_3_ and CH_2_, respectively. The C=C vibrations were shown around 1680 cm^−1^. Band at 1460 and 1390 cm^−1^ was assigned for C-H bending and 1050 for C-O stretching of OH [18]. The MS spectrum of the compound (C_30_H_50_O) showed parent molecular ion [M^+^] peak at m/z 426 (35%) for (C_30_H_50_O^+^). The ion peak at m/z 411 (100%) corresponds to the molecular formula ion (C_29_ H_47_O^+^), while the ion peak at m/z 393 (62%) corresponds to the molecular formula ion (C_29_H_45_^+^) as a result of the loss of methyl group and water [19, 20].

The identification of the compound was performed by comparing its spectral data, including ^1^H, ^13^C NMR, with Shin et al. [20]. The ^1^H NMR spectrum revealed the presence of seven tertiary methyl protons at *δ*0.75 (H_18_), 0.95 (H_19_), 1.68 (H_26_), 1.60 (H_27_), 1.00 (H_28_), 0.80 (H_29_), and 0.85 (H_30_). A doublet of three protons at *δ*0.88 with *J* = 4 corresponds to (H_21_), and the triplet of one proton at *δ*5.11 with *J* = 8 Hz corresponds to H_24_. The proton appeared as a doublet of the doublet with *J* = 4 and 12 Hz at *δ*3.25, 3.22. The identification of the compound was performed by comparing its spectral data including ^1^H, ^13^C NMR with Shin et al., 2000 [20]. ^1^H NMR spectrum revealed the presence of seven tertiary methyl protons at *δ*0.75 (H_18_), 0.95 (H_19_), 1.68 (H_26_), 1.60 (H_27_), 1.00 (H_28_), 0.80 (H_29_), and 0.85 (H30). A doublet of three protons at *δ*0.88 with *J* = 4 corresponds to (H21), and the triplet of one proton at *δ*5.11 with *J* = 8 Hz corresponds to H_24_. The proton appeared as a doublet of the doublet with *J* = 4 and 12 Hz at *δ*3.25, 3.22 to H-_3_.

The ^13^C NMR spectrum showed seven methyl groups at *δ*15.62 (C_−18_), 20.15 (C-_19_), 18.92 (C-_26_), 27.67 (C-_27_), 24.77 (C-_28_), 28.05 (C-_29_), and 15.53 (C-_30_). The deshielded signal at *δ*79.01 was due to the presence of hydroxyl group at C-_3_. The signals at *δ*133.55 (C_8_), 134.04 (C_9_), 125.22 (C_24_), and 130.87 (C_25_) were due to the presence of a double bond. The comparison of the spectral data with those reported previously led to the proposal of the lanosterol structure. Lanosterol (lanosta-8,24-dien-3-ol)-type triterpene with a cyclopentanol partial structure in the side chain has not been reported from *E. arbuscula.*

Compound **(2)**: The melting point (215–217°C) was in agreement with the previously published data of Lupeol [21]. The compound showed a violet spot visible on TLC when sprayed by vanillin sulfuric acid indicating that this compound could be a sterol [17]. The IR spectrum showed a broad strong band at 3397 cm^−1^, indicating the presence of OH. The two bands at 2941 and 2857 cm^−1^ indicated the CH stretching of CH_3_ and CH_2_, respectively. The C=C vibration was shown around 1638 cm^−1^. Bands at 1454 and 1379 (C-H bending) and 1043 cm^−1^ indicated C-O stretching of OH [21].

The MS spectrum (C_30_H_50_O) showed parent molecular ion [M^+^] peak at m/z 426 (35%) corresponding to C_30_H_50_O^+^. The ion peak at m/z 411 (19.7%) corresponds to molecular formula ion of C_29_H_47_O^+^ as a result of the loss of the methyl group. Due to the broken side chains of C_13_ and C_9_, they can easily decompose into other fragment ions with lower m/z 207 (50.3%), 189 (70%), 175 (30.2%), and 95 (99%), corresponding to the molecular formula ion (C_14_H_23_O^+^), (C_14_H_21_^+^), (C_13_H_19_^+^), and (C_7_H_11_^+^), respectively [22].

The identification of this compound was performed by comparing its spectral data including ^1^H and ^13^C NMR with those published data with Htay et al. [23]. The ^1^H NMR spectrum revealed the presence of seven signals singlet for tertiary methyl protons at *δ* 0.96 (H_23_), 0.72 (H_24_), 0.88 (H_25_), 1.32 (H_26_), 0.90 (H_27_), 0.76 (H_28_), and 1.61 (H_30_). Multiples of one proton at *δ* 2.36 correspond to H19. The H-3 proton appeared as a doublet of the doublet with *J* = 4.1 and 10.9 Hz at *δ*3.10, 3.12. The doublet at *δ* 4.50 and 4.62 with *J* = 1.4 Hz due to two methylene protons (H29) is a characteristic of lupeol.

The ^13^C NMR spectrum showed seven methyl groups at *δ* 27.99 (C- _23_), 15.38 (C-_24_), 16.13 (C-_25_), 15.99 (C-_26_), 14.56 (C-_27_), 18.01 (C-_28_), and 19.32 (C-_30_). The deshielded signals at *δ* 79.02 were due to the presence of the hydroxyl group at C-3. The signals at *δ*150.98 (C_20_) and 109.32 (C_29_) were due to the presence of a double bond. The compound was identified as lupeol, and its spectral data showed good agreement with those reported in the literature. Lupeol (lup-20(29)-en-3*β*-ol) is a pentacyclic triterpene. It has not been reported for *E. arbuscula.*

### 4.1. Antibacterial Activity

The antibacterial activity varies with the species of the plant and the plant material. *E. thymifolia* showed a less potent antibacterial response compared to *E. hirta* [24]. On the other hand, *E. cotinifolia* was found to be resistant to antibacterial activity against any bacteria tested [25]. Recently, researchers shown that the multi-resistant bacterial strains have increased dramatically and thus the treatment of several infections has become very difficult to reduce the therapeutic options [26].

### 4.2. Antioxidant Activity

The findings of our study revealed differences in the antioxidant activities of plant extracts and isolation compounds. The highest activity was obtained from the ethyl acetate extract with an IC_50_ value of (±13.55 *μ*g/mL), which was comparable to ascorbic acid (IC_50_ ± 4.09 *μ*g/mL), followed by the chloroform extract (IC_50_ ± 21.87 *μ*g/mL). The highest activity of the ethyl acetate and chloroform extracts could be due to the synergistic action of the compounds contained, rather than a single chemical substance. Previous studies have reported on the antioxidant activity of various Euphorbia species, including *E. acanthamnos*, *E. macroclada*, *E. rigida*, and *E. heyneana* [27, 28].

## 5. Conclusion

The ethyl acetate extract of *Euphorbia arbuscula* exhibited the highest antioxidant activity, which was comparable to that of ascorbic acid, followed by the chloroform extract. Antioxidants play a crucial role in protecting health by reducing the risk of chronic diseases, including cancer and heart disease. Further testing of the ethyl acetate extract may yield additional bioactivity, and there is potential for the isolation of bioactive compounds. In addition, more studies are needed to determine the toxicological and pharmacological properties of this plant species. This research provides opportunities for future work, as many compounds have not yet been identified and isolated.

## Figures and Tables

**Table 1:** The overall yield and percentage of stem latex extracts of *E. arbuscula*.

Latex extracts	Character	Weight (g)	Percentage of yield (%) w/A	Percentage of yield (%) w/B
Ethanol	Yellowish brown semisolid	309.05	80.48	—
First oil layer	Yellow semisolid	222.09	57.83	71.86
Second oil layer	Yellow semisolid	42.07	10.95	13.61
Precipitate from	Yellow powder	4.77	1.24	1.54

*Methanol layer*				
Methanol	Brown semisolid	38.12	9.92	12.33
n-Hexane	Yellow powder	15.54	4.04	5.02
Chloroform	Brown semisolid	1.85	0.48	0.59
Ethyl acetate	Dark brown powder	1.08	0.28	0.34
Butanol	Brown semisolid	13.89	3.61	4.48

A: weight of the dried stem latex (384 g) of *E. arbuscula* and B: weight of the crude ethanol extract (309.05 g) stem latex of *E. arbuscula*.

**Table 2:** Phytochemical screening of stem latex extracts of *E. arbuscula*.

Phytochemical constituent	Crude extracts				
	Total MeOH	n-Hex	CHCl_3_	EtOAc	n-BuOH
Terpenoids (Salkowski test)	+	+	+	−	−
Alkaloids					
Mayer's test	+	−	−	+	+
Wagner's test	+	−	−	+	+
Flavonoids	+	−	+	+	−
Tannins	+	+	+	+	+
Phenols	+	+	+	+	+
Saponins	−	−	−	+	+
Glycoside	−	−	−	−	−
Cardiac glycoside	+	−	+	+	−
Steroids	+	+	+	−	−

+: presence; −: nonpresence; MeOH: methanol; n-Hex: n-hexane; CHCl_3_: chloroform; EtOAc: ethyl acetate; n-BuOH: n-butanol.

**Table 3:** List of chemical composition present in oily layer obtained from extract of stem latex of *E. arbuscula* by GC-MS.

No.	RT	Name of compound	MF	MW	% peak area	Type
1	32.96	Diisooctyl phthalate	C_24_H_38_O_4_	390	9.93	Ester
2	40.74	Lanosta-8,24-dien-3-one	C_30_H_48_O	424	0.77	Triterpene
3	40.83	Acetic acid, 17-(1,5-dimethylhex-4-enyl)-4,4,8,10,14-pentamethyl-2,3,4,5,6,7,8,9,10,11,12,14,15,16-tetradecahydro-1H-cyclopenta[a]phenanthrene	C_32_H_52_O_2_	468	1.79	Triterpene
4	41.25	Lanosterol	C_30_H_50_O	426	51.98	Triterpene
5	41.73	Ergosta-8,24(28)-dien-3-ol 4,14-dimethyl	C_30_H_50_O	426	17.82	Triterpene
6	41.99	Lupenyl acetate	C_32_H_52_O_2_	468	2.15	Triterpene
7	42.26	Cycloartenol	C_30_H_50_O	426	2.90	Triterpene
8	42.86	Lupeol	C_30_H_50_O	426	9.86	Triterpene

**Table 4:** ^13^C NMR spectroscopic data for compound (1) lanosterol and compound (2) lupeol CDCl_3_a.

Lanosterol *δ* C compound (1)		Lupeol *δ* C compound (2)	
Position		Position
1	35.26	1	38.74
2	27.95	2	27.45
3	79.01	3	79.02
4	38.94	4	38.74
5	50.97	5	55.32
6	24.77	6	18.33
7	28.14	7	34.31
8	133.55	8	40.86
9	134.04	9	50.46
10	37.27	10	37.20
11	21.53	11	20.94
12	28.14	12	25.15
13	44.12	13	38.08
14	50.03	14	42.83
15	31.59	15	27.44
16	30.90	16	35.59
17	49.64	17	43.03
18	15.62	18	48.33
19	20.15	19	48.01
20	35.88	20	150.98
21	18.95	21	29.86
22	35.43	22	40.01
23	25.74	23	27.99
24	125.22	24	15.38
25	130.87	25	16.13
26	18.92	26	15.99
27	27.67	27	14.56
28	24.47	28	18.01
29	28.05	29	109.32
30	15.53	30	19.32

a: chemical shift values are given in ppm.

## Data Availability

All data are available upon request to the corresponding author.
